# Editorial: Trends in dengue evolution, immune pathogenesis, and pathology

**DOI:** 10.3389/fcimb.2023.1210316

**Published:** 2023-05-25

**Authors:** S Gowri Sankar, A Alwin Prem Anand, Balaji Chattopadhyay

**Affiliations:** ^1^ Department of Molecular Biology, Indian Council of Medical Research (ICMR)-Vector Control Research Center - Field Station, Madurai, India; ^2^ Institute of Clinical Anatomy and Cell Analysis, University of Tübingen, Tübingen, Germany; ^3^ Trivedi School of Biosciences, Ashoka University, Sonepat, India

**Keywords:** dengue phylogeny, innate immunity, adaptive immunity, T cell immune response, B cell immune response

Dengue is an arthropod borne viral infection that is listed as a neglected tropical disease (Wilder-Smith et al., 2019). It is one of the major health threats globally and has experienced a massive increase in incidents over the past half century. However, no successful vaccine has been developed yet. All candidate vaccines show unpredictable complexity, including that vaccine efficacy is dependent on serotype, age, and serostatus i.e., it increases risk for seronegative recipients (Capeding et al., 2014; Sridhar et al., 2018). In order to understand dengue pathology and pathogenesis, it is of critical importance to investigate dengue viral evolution and its immune mediated pathogenesis. In this direction, the current Research Topic “*Trends in Dengue Evolution, Immune Pathogenesis, and Pathology*” and its collection of six articles provide significant insights into dengue infection, particularly towards immunity, pathogenesis, epidemiology, and evolution of dengue.

Han et al. used a metagenomic approach to generate entire genomes from four isolates and investigated the evolution of Dengue virus during an outbreak in Wenzhou, Southeast China. All the sequences were observed to be part of the DENV1 genotype. Two samples clustered with sequences reported from Singapore and Vietnam, and the other two genomes formed a sister clade basal to most other DENV 1 sequences. Analyses revealed the presence of positive selection, multiple recombination events, and changes in the head and tail of the 3’UTR in the local strains sequenced as part of the study compared to the reference sequence.

Enteric dysbiosis has been discovered in viral infections including influenza virus (Yildiz et al., 2018), hepatitis C virus (Inoue et al., 2018), and COVID-19 (Gu et al., 2020). Enteric dysbiosis leads to leaky gut syndrome, where increased intestinal permeability results in translocation of gut microbiota into blood circulation. Recently, a study has shown leaky gut syndrome is associated with endotoxemia in severe dengue patients (Chancharoenthana et al., 2021). Chancharoenthana et al. succinctly put forth a rare demonstration of the movement of bacteria from gut to blood stream during dengue infection. Using an NGS-based metabarcoding strategy, they identified bacteria to the phylum level and observed an association with increased abundance of *Bacteroidetes* and *Escherichia* spp. with severity in infection when compared to the control.

Cross-reactive immunity among flaviviruses is commonly observed due to their antigenic similarities (De Madrid and Porterfield, 1974; Calisher et al., 1989; Rathore and St John, 2020). Highly conserved epitopes hamper the diagnosis, treatment, and prevention of flaviviruses especially in DENV (dengue virus) serotypes, ZIKV (zika virus), WNV (west Nile virus), and JEV (Japanese encephalitis virus). The review by Chan et al. describes different serological tests, such as neutralization tests, enzyme-linked immunosorbent assay, hemagglutination-inhibition test, Western blot test, and immunofluorescence assay. The in-depth review provides the current concept of flavivirus cross-reactivity and finally identifies neutralization tests as the gold standard to eliminate cross-reactivity among flaviviruses.

It is well-known that DENV and ZIKV belong to same family, *Flaviviridae*, and several studies show cross-reactivity between DENV and ZIKV sera (Dejnirattisai et al., 2016; Paul et al., 2016; Priyamvada et al., 2016; Montoya et al., 2018). Sekaran et al. reviewed and summarized the host immune responses including innate and adaptive against DENV and ZIKV infections as well as cross-reactivity between DENV and ZIKV, and stressed the necessity to understand the mechanism of T cell subset for disease prevention.

A balanced innate immune response is essential for the control of DENV infection, as viruses continuously evolve to circumvent immune response. Lee et al. reviewed how DENV evade host immune response. Interestingly, DENV engage in RNA modifications as a mode for immune evasion by DENV that includes 1) camouflaging viral RNAs as cellular mRNAs, 2) increasing subgenomic flavivirus RNA (sfRNA) that binds and deubiquitylates TRIM25, further preventing activation of RIG-1-mediated IFN signalling and 3) providing stability of sfRNA by pseudoknots in 3’UTR that prevent degradation by cellular RNase.

In dengue, immune evasion is mediated by nonstructural proteins including NS1 (Avirutnan et al., 2010; Avirutnan et al., 2011; Glasner et al., 2018), NS2B (Aguirre et al., 2017), NS3 (Chan and Gack, 2016), NS2A, NS4A, NS4B, and NS5 (Morrison et al., 2012; Castillo Ramirez and Urcuqui-Inchima, 2015). In this collection, Udawatte et al. report that DENV NS3 protein targets receptor interacting protein kinase I (RIPK1), a central mediator of inflammation and cell death, and decreases intracellular RIPK1 levels during DENV infection. The interaction of NS3 with RIPK1 results in the inhibition of NF-kB activation in response to TNFR or TLR3 stimulation. This is an interesting find, adding to the information on the important role of NS3 in immune evasion.

## Author contributions

All authors listed have made a substantial, direct, and intellectual contribution to the work and approved it for publication.
